# PERSPECTIVES OF THE HOME HEALTH WORKFORCE ON TRAINING

**DOI:** 10.1093/geroni/igad104.2396

**Published:** 2023-12-21

**Authors:** Elise Valdes

**Affiliations:** Relias Inc., Tampa, Florida, United States

## Abstract

As more older adults desire to age in place, home health workers are becoming a vital component of the healthcare workforce. The last large scale nationwide survey among home health workers was conducted in 2007. There has been a variety of research in this area since then; however, the majority of studies are small and qualitative in nature which may limit their generalizability. Understanding the opinions and perspectives of home health workers is vital to retaining this important part of the healthcare workforce. For the current study a broad sample of home health workers (n=1446) including home health aides, hospice health aides, personal care aides, CNAs, LVN/LPNs, and RNs completed an online self-report survey on how well their training prepared them for practice. Results indicated that most hospice health aides felt their training well prepared them for practice (80.3%), while only 56.6% of RNs reported that they felt well prepared. We also explored how prepared respondents felt on a variety of topics from patient care skills to dementia care to end of life skills. Again, hospice health aides reported the highest average scores for receiving excellent training across all topics (61.5%) and RNs reported the lowest average scores (42.6%). Feeling unprepared for a job, may lead to lower job satisfaction and higher turnover. Therefore, understanding how well-prepared home health workers feel is key to retaining this segment of the workforce. Future research should explore how to improve this training and preparation across all home health workers but particularly among RNs.

